# Pairwise interact-and-imitate dynamics

**DOI:** 10.1038/s41598-021-92512-5

**Published:** 2021-06-24

**Authors:** Ennio Bilancini, Leonardo Boncinelli, Nicola Campigotto

**Affiliations:** 1 Laboratory for the Analysis of CompleX Economic Systems, IMT School for Advanced Studies Lucca, 55100 Lucca, Italy; 2 Department of Economics and Management, University of Florence, 50127 Florence, Italy; 3 Department of Economics and Management, University of Pisa, 56124 Pisa, Italy

**Keywords:** Population dynamics, Social evolution, Human behaviour

## Abstract

This paper introduces and studies a class of evolutionary dynamics—pairwise interact-and-imitate dynamics (PIID)—in which agents are matched in pairs, engage in a symmetric game, and imitate the opponent with a probability that depends on the difference in their payoffs. We provide a condition on the underlying game, named supremacy, and show that the population state in which all agents play the supreme strategy is globally asymptotically stable. We extend the framework to allow for payoff uncertainty, and check the robustness of our results to the introduction of some heterogeneity in the revision protocol followed by agents. Finally, we show that PIID can allow the survival of strictly dominated strategies, leads to the emergence of inefficient conventions in social dilemmas, and makes assortment ineffective in promoting cooperation.

## Introduction

In evolutionary game-theoretic models, it is standard practice to assume that agents make decisions according to short-sighted adaptive rules. These include avoidance of strategies that performed poorly in the past, best response to the empirical distribution of opponents’ strategies, and imitation of successful peers^1,2^. The last has been shown to be common in both humans and animals, and is generally recognized as a cognitively parsimonious social heuristic^3–5^. An important aspect of imitative dynamics is the relation between the structure of interactions and agents’ reference groups. The interaction structure specifies how agents are matched, e.g. in a purely random manner or assortatively in some respect^6–11^; an individual’s reference group consists instead of those agents whom that individual observes and takes as a reference for comparison purposes. This paper examines the case where agents compare their payoff to that of their opponent, obtaining clear-cut and perhaps surprising results in a variety of games. In doing so, it shows that the interplay of interaction structure and reference groups, which so far has received little attention in the literature, plays a fundamental role in determining evolutionary outcomes.

Distinctions among imitative rules can be made as to what drives behavior, who is imitated, and how much information is needed for decision making^12,13^. For example, rules of the kind ‘copy the first person you see’ make actions depend only on their popularity, whereas other rules consider actions to be a function of observed payoffs. The target of comparisons may consist of either a single agent or a (possibly large) group of individuals, and information requirements can range from very low to extremely high levels, yielding a wide range of different behavioral rules^14–21^. Often, these rules treat interaction structure and reference groups as separate entities: whenever an agent receives a revision opportunity, she randomly selects another individual as reference, observes this individual’s strategy, and switches to it with a probability that depends on relative payoffs^22–24^. This is most plausible in the case of games against nature or when agents cannot observe their opponents’ payoff. However, cases also exist in which the decoupling of interaction structure and reference groups does not hold, as often people can only observe, and act upon, the behavior of those with whom they interact. This idea is recurrent in the literature on games on networks, where typically agents play with and imitate their nearest or next-nearest neighbors^25–30^.

Building on this insight, this paper introduces and studies a class of evolutionary dynamics in which interaction structure and reference groups overlap, that is, where those whom one interacts with are also those with whom she compares herself. When given a revision opportunity, an agent playing strategy *i* against an opponent playing strategy *j* will switch to *j* with positive probability if the payoff from *j* against *i* is greater than the payoff from *i* against *j*. We name this revision protocol *Pairwise Interact-and-Imitate*. Intuitively, this appears to be a reasonable criterion for strategy updating in situations where interacting with another agent suffices to make that agent salient as a comparison reference, which may occur, for instance, when interaction and observation opportunities are constrained by the same factors, be them physical, social or cultural. In such cases an overlap between interaction structure and reference groups is established indirectly, as the result of both interaction and observation being determined by the same factors.

Our work is close in spirit to pairwise comparative models of traffic dynamics, where changes from one route to another occur at a frequency that depends on differences in traveling costs^31^. It is also related to local replicator dynamics^32^, in which agents are uniformly matched at random in groups of size *n*, engage in pairwise interactions with members of their group, and imitate each other depending on the difference in their payoffs; when $n = 2$ these models yield a Pairwise Interact-and-Imitate dynamic with uniform random matching (while here we also consider matching processes that are not uniformly random).

The purpose of our paper is twofold. First, we introduce the Pairwise Interact-and-Imitate revision protocol and study the resulting dynamics in symmetric games. We give a condition on the stage game, named supremacy, and show that the population state in which all agents choose the supreme strategy is globally asymptotically stable. Roughly speaking, a strategy is supreme if it always yields a payoff higher than the payoff received by an opponent playing a different strategy. We then generalize the framework to allow for payoff uncertainty, we check the robustness of our results to the introduction of some heterogeneity in revision protocols, and we show that PIID can allow the survival of strictly dominated strategies. Second, we apply the revision protocol to social dilemmas, showing that PIID causes the emergence of inefficient conventions and makes assortment ineffective in facilitating cooperation.

## Results

### The model

Consider a unit-mass population of agents who repeatedly interact in pairs to play a symmetric stage game. The set of strategies available to each agent is finite and denoted by $S \equiv \{1, \ldots , n\}$. A population state is a vector $x \in X \equiv \{x \in {\mathbb{R}}^n_+: \sum _{i \in S} x_i = 1\}$, with $x_i$ the fraction of the population playing strategy $i \in S$. Payoffs are described by a function $F: S \times S \rightarrow {\mathbb{R}}$, where *F*(*i*, *j*) is the payoff received by an agent playing strategy *i* when the opponent plays strategy *j*. As a shorthand, we refer to an undirected pair of individuals, one playing *i* and the other playing *j*, as an *ij* pair. The set of all possible undirected pairs is denoted by $\mathscr {P}$.

The interaction structure is modeled as a function $$p : X \times \mathscr {P} \rightarrow \left[ 0, 1/2 \right] $$ subject to $$\sum _{ij \in \mathscr {P}} p_{ij}(x)=1/2$$ (since the mass of pairs is half the mass of agents), with $$p_{ij}(x)$$ indicating the mass of *ij* pairs formed in state *x*. Note that the mass of *ij* pairs can never exceed $$\min \{x_i,x_j\}$$, that is, $$p_{ij}(x) \le \min \{x_i,x_j\}$$ for all *x*. We assume that *p* is continuous in *X*, and that $$p_{ij}(x) > 0$$ if and only if $$x_i > 0$$ and $$x_j > 0 $$—meaning that the probability of an *ij* pair being formed is strictly positive if and only if strategies *i* and *j* are played by someone. In the case of uniform random matching, $$p_{ii} = x_i^2/2$$ and $$p_{ij} = x_i x_j$$ for any *i* and $$j \ne i$$.

The revision protocol is modeled as a function $\phi : X \times S \times S \rightarrow [-1,1]$, where $\phi _{ij}(x) \in [-1,1]$ is the probability that an *ij* pair will turn into an *ii* pair minus the probability that it will turn into a *jj* pair, conditional on the population state being *x* and an *ij* pair being formed. We assume that $\phi$ is continuous in *X*. We note that by construction $\phi _{ij}=-\phi _{ji}$ for all $i,j \in S$, and hence $\phi _{ii}=0$ for all $i \in S$. Our main assumption on the revision protocol is the following, which is met, among others, by pairwise proportional imitative and imitate-if-better rules^22^.

#### **Assumption 1**

For every $x \in X$, $\phi _{ij}(x) > 0$ if $F(i,j) > F(j,i)$.

In what follows we consider a dynamical system in continuous time with state space *X*, characterized by the following equation of motion.

#### **Definition 1**

(*Pairwise interact-and-imitate dynamics—PIID*) For every $x \in X$ and every $i \in S$:

1 $$\begin{aligned} \dot{x}_i = \sum _{j \in S} p_{ij}(x) \phi _{ij}(x). \end{aligned}$$

### Main findings

#### Global asymptotic convergence

In any purely imitative dynamics, if $x_i(t)=0$, then $x_i(t^{\prime})=0$ for every $t^{\prime} > t$. This implies that we cannot hope for global asymptotic convergence in a strict sense. Thus, to assess convergence towards a certain state *x* in a meaningful way, we restrict our attention to those states where all strategies that have positive frequency in *x* have positive frequency as well. We denote by $X_x$ the set of states whose support contains the support of *x*.

##### **Definition 2**

(*Supremacy*) Strategy $i\in S$ is supreme if $F(i,j)>F(j,i)$ for every $j \in S \setminus \{i\}$.

We note that under PIID, the concept of supremacy is closely related to that of asymmetry^33,34^, in that $F(i,j) > F(j,i)$ implies that agents can only switch from strategy *j* to strategy *i*.

##### **Proposition 1**

*If* $i \in S$ *is a supreme strategy, then state* $x^* \equiv \left\{ x \in X : x_i = 1 \right\}$ *is globally asymptotically stable for the dynamical system with state space* $X_{x^*}$ *and PIID as equation of motion*.

#### Relation to replicator dynamics

To further characterize the dynamics induced by the pairwise interact-and-imitate protocol, we make two additional assumptions. First, matching is uniformly random, meaning that everyone in the population has the same probability of interacting with everyone else; formally, $p_{ii} = x_i^2/2$ and $p_{ij} = x_i x_j$ for all *i* and $j \ne i$. Second, the probability that an agent has to imitate the opponent is proportional to the difference in their payoffs if the opponent’s payoff exceeds her own, and is zero otherwise. As a consequence, $\phi _{ij} = F(i,j) - F(j,i)$ up to a proportionality factor. Let

- $$F \left( i, x \right) :=\sum _j x_j F \left( i, j \right) $$,
- $$F \left( x, i \right) :=\sum _j x_j F \left( j, i \right) $$, and
- $$ F \left( x, x \right) :=\sum _i \sum _j x_i x_j F \left( i, j \right) $$.

Under these assumptions, at any point in time, the motion of $x_i$ is described by:

2 $$\begin{aligned} \dot{x}_i&= \sum _{j \ne i} x_j x_i \left[ F \left( i, j \right) - F \left( j, i \right) \right] = x_i \sum _{j} x_j \left[ F \left( i, j \right) - F \left( j, i \right) \right] \nonumber \\&= x_i \left[ F \left( i, x \right) - F \left( x, i \right) \right] , \end{aligned}$$

which is a modified replicator equation. According to (2), for every strategy *i* chosen by one or more agents in the population, the rate of growth of the fraction of *i*-players, $\dot{x}_i / x_i$, equals the difference between the expected payoff from playing *i* in state *x* and the average payoff received by those who are matched against an agent playing *i*. In contrast, under standard replicator dynamics^35^, the fraction of agents playing *i* varies depending on the excess payoff of *i* with respect to the current average payoff in the whole population, i.e., $\dot{x}_i = x_i \left[ F \left( i, x \right) - F \left( x, x \right) \right]$.

A noteworthy feature of replicator dynamics is that they are always payoff monotone: for any $i,j \in S$, the proportions of agents playing *i* and *j* grow at rates that are ordered in the same way as the expected payoffs from the two strategies^36^. In the case of PIID, this result fails.

##### **Proposition 2**

*Pairwise-Interact-and-Imitate dynamics need not satisfy payoff monotonicity*.

**Figure 1:**
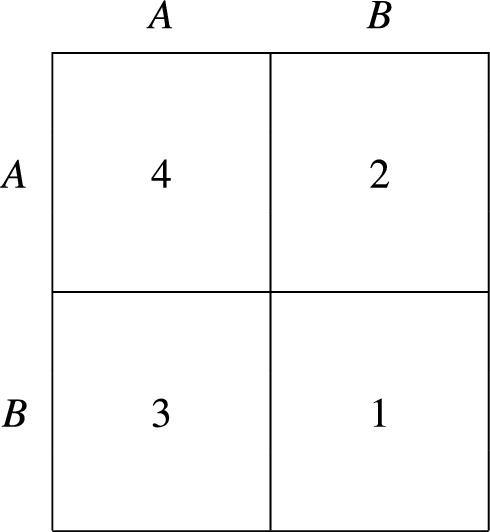
A game where the supreme strategy is strictly dominated.

To verify this, it is sufficient to consider any symmetric $2 \times 2$ game where $F \left( i, j \right) > F \left( j, i \right)$ but $F \left( j, x \right) > F \left( i, x \right)$ for some $x \in X$, meaning that *i* is the supreme strategy but *j* yields a higher expected payoff in state *x*. See Fig. 1 for an example where, in the case of uniform random matching, the above inequalities hold for any *x*; if strategies are updated according to the interact-and-imitate protocol, then this game only admits switches from *i* to *j*, therefore violating payoff monotonicity. Proposition 2 can have important consequences, including the survival of pure strategies that are strictly dominated.

#### Survival of strictly dominated strategies

An recurring topic in evolutionary game theory is to what extent does support exist for the idea that strictly dominated strategies will not be played. It has been shown that if strategy *i* does not survive the iterated elimination of pure strategies strictly dominated by other pure strategies, then the fraction of the population playing *i* will converge to zero in all payoff monotone dynamics^37,38^. This result does not hold in our case, as PIID is not payoff monotone.

More precisely, under PIID, a strictly dominated strategy may be supreme and, therefore, not only survive but even end up being adopted by the whole population. This suggests that from an evolutionary perspective, support for the elimination of dominated strategies may be weaker than is often thought. Our result contributes to the literature on the conditions under which evolutionary dynamics fail to eliminate strictly dominated strategies in some games, examining a case which has not yet been studied^39^.

To see that a strictly dominated strategy may be supreme, consider the simple example shown in Fig. 1. Here each agent has a strictly dominant strategy to play *A*; however, since the payoff from playing *B* against *A* exceeds that from playing *A* against *B*, strategy *B* is supreme. Thus, by Proposition 1, the population state in which all agents choose *B* is globally asymptotically stable.

Figure 1 can also be used to comment on the relation between a supreme strategy and an evolutionary stable strategy, which is a widely used concept in evolutionary game theory^40,41^. Indeed, while *B* is the supreme strategy, *A* is the unique evolutionary stable strategy because it is strictly dominant. However, if *F*(*B*, *A*) were reduced below 2, holding everything else constant, then *B* would become both supreme and evolutionary stable. We therefore conclude that no particular relation holds between evolutionary stability and supremacy: neither one property implies the other, nor are they incompatible.

### Applications

Having obtained general results for the class of finite symmetric games, we now restrict the discussion to the evolution of behavior in social dilemmas. We show that if the conditions of Proposition 1 are met, then inefficient conventions emerge in the Prisoner’s Dilemma, Stag Hunt, Minimum Effort, and Hawk–Dove games. Furthermore, this result holds both without and with the assumption that agents interact assortatively.

#### Ineffectiveness of assortment

**Figure 2:**
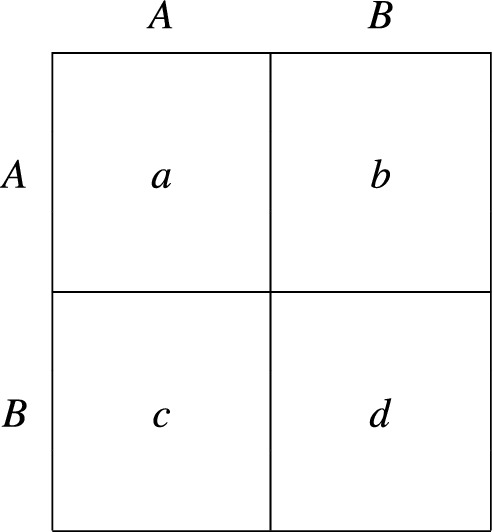
A $$2 \times 2$$ stage game.

Consider the $2 \times 2$ game represented in Fig. 2. If $c> a> d > b$, then mutual cooperation is Pareto superior to mutual defection but agents have a dominant strategy to defect. The resulting stage game is the Prisoner’s Dilemma, whose unique Nash equilibrium is (*B*, *B*). Moreover, since $F (B,A) > F(A,B)$, *B* is the supreme strategy and the population state in which all agents defect is globally asymptotically stable.

We stress that defection emerges in the long run for every matching rule satisfying our assumptions, and therefore also in the case of assortative interactions. Assortment reflects the tendency of similar people to clump together, and can play an important role in the evolution of cooperation^42–45^. Intuitively, when agents meet assortatively, the risk of cooperating in a social dilemma may be offset by a higher probability of playing against other cooperators. However, under PIID, this is not the case: the decision whether to adopt a strategy or not is independent of expected payoffs, and like-with-like interactions have no effect except to reduce the frequency of switches from *A* to *B*.

#### Emergence of the maximin convention

If $a> c > b$, $a > d$ and $d > b$, then the game in Fig. 2 becomes a Stag Hunt game, which contrasts risky cooperation and safe individualism. The payoffs are such that both $\left( A, A\right)$ and $\left( B, B\right)$ are strict Nash equilibria, that $\left( A, A\right)$ is Pareto superior to $\left( B, B\right)$, and that *B* is the maximin strategy, i.e., the strategy which maximizes the minimum payoff an agent could possibly receive. We also assume that $a + c \ne c + d$, so that one of *A* and *B* is risk dominant^46^. If $a + b > c + d$, then *A* (Stag) is both payoff and risk dominant. When the opposite inequality holds, the risk dominant strategy is *B* (Hare).

Since $F (B,A) > F(A,B)$, *B* is supreme independently of whether or not it is risk dominant to cooperate. This can result in large inefficiencies because, in the long run, the process will converge to the state in which all agents play the riskless strategy regardless of how rewarding social coordination is. As in the case of the Prisoner’s Dilemma, this holds for all matching rules satisfying our assumptions.

#### Evolution of effort exertion

In a minimum effort game, agents simultaneously choose a strategy *i*, usually interpreted as a costly effort level, from a finite subset *S* of ${\mathbb{R}}$. An agent’s payoff depends on her own effort and on the minimum effort in the pair:

$$\begin{aligned} F \left( i, j \right) = \alpha \min \left\{ i, j \right\} - \beta i , \end{aligned}$$

where $\beta > 0$ and $\alpha > \beta$ are the cost and benefit of effort, respectively. From a strategic viewpoint, this game can be seen as an extension of the Stag Hunt to cases where there are more than two actions. The best response to a choice of *j* by the opponent is to choose *j* as well, and coordinating on any common effort level gives a Nash equilibrium. Nash outcomes can be Pareto-ranked, with the highest-effort equilibrium being the best possible outcome for all agents. Thus, choosing a high *i* is rationalizable and potentially rewarding but may also result in a waste of effort.

Under PIID, any $i > j$ implies $\phi _{ij} < 0$ by Assumption 1, meaning that agents will tend to imitate the opponent when the opponent’s effort is lower than their own. The supreme strategy is therefore to exert as little effort as possible, and the population state in which all agents choose the minimum effort level is the unique globally asymptotically stable state.

#### Emergence of aggressive behavior

Consider again the payoff matrix shown in Fig. 2. If $c> a> b > d$, then the stage game is a Hawk–Dove game, which is often used to model the evolution of aggressive and sharing behaviors. Interactions can be framed as disputes over a contested resource. When two Doves (who play *A*) meet, they share the resource equally, whereas two Hawks (who play *B*) engage in a fight and suffer a cost. Moreover, when a Dove meets a Hawk, the latter takes the entire prize. Again we have that $F (A,B) < F(B,A)$, implying that *B* is the supreme strategy and that the state where all agents play Hawk is the sole asymptotically stable state.

The inefficiency that characterizes the (*B*, *B*) equilibrium in the Hawk–Dove game arises from the cost that Hawks impose on one another. This can be viewed as stemming from the fact that neither agent owns the resource prior to the interaction or cares about property. A way to overcome this problem may be to introduce a strategy associated with respect for ownership rights, the Bourgeois, who behaves as a Dove or Hawk depending on whether or not the opponent owns the resource^41^. If we make the standard assumption that each member of a pair has a probability of 1/2 to be an owner, then in all interactions where a Bourgeois is involved there is a 50 percent chance that she will behave hawkishly (i.e., fight for control over the resource) and a 50 percent chance that she will act as a Dove.

**Figure 3:**
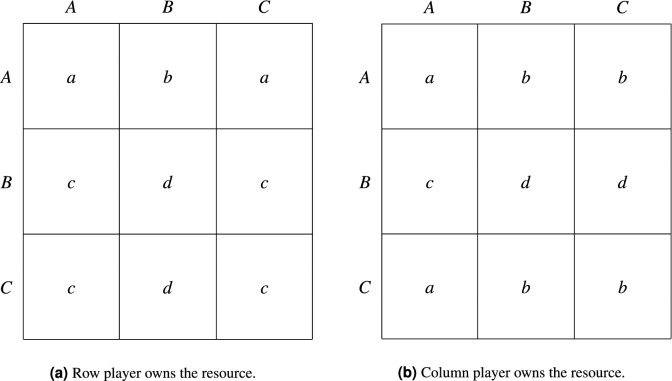
The Hawk–Dove–Bourgeois game.

Let *R* and *C* denote the agent chosen as row and column player, respectively, and let $\omega _R$ and $\omega _C$ be the states of the world in which *R* and *C* owns the resource. The payoffs of the resulting Hawk–Dove–Bourgeois game are shown in Fig. 3. If agents behave as expected payoff maximizers, then All Bourgeois can be singled out as the unique asymptotically stable state. Under PIID, this is not so; depending on who owns the resource, an agent playing *C* against an opponent playing *B* may either fight or avoid conflict and let the opponent have the prize. It is easy to see that $F \left( C, B \mid \omega _R \right) = F \left( B,C \mid \omega _C \right) = d$, meaning that the payoff from playing *C* against *B*, conditional on owning the resource, equals the payoff from playing *B* against *C* conditional on not being an owner. In contrast, the payoff from playing *C* against *B*, conditional on not owning the resource, is always worse than that of the opponent, i.e., $F \left( C, B \mid \omega _C \right) = b < c = F \left( B, C \mid \omega _R \right)$. Thus, in every state of the world, *B* (Hawk) yields a payoff that is greater or equal to that from *C* (Bourgeois). Moreover, since $F \left( B,A \right) > F \left( A, B \right)$ in both states of the world, strategy *B* is weakly supreme by Definition 4, and play unfolds as an escalation of hawkishness and fights.

## Discussion

We have studied a novel class of evolutionary dynamics, named pairwise interact-and-imitate dynamics, in which agents choose whether or not to change strategy by comparing their payoff with that of their opponent. Our main result is that under PIID, if there exists a supreme strategy (that is, a strategy that always yields a payoff higher than the payoff received by an opponent playing a different strategy), then the state in which the whole population chooses the supreme strategy is globally asymptotically stable. Importantly, the supreme strategy may be a dominated strategy, and the strategy profile played in the asymptotically stable state may not be a Nash equilibrium.

Under PIID, externalities have an important role. Whenever a strategy, say *i*, generates an increase (decrease) in the payoff of an opponent playing a different strategy, say *j*, it is more (less) likely that *i* will be updated in favor of *j*. Moreover, this is so regardless of the payoff received when using the same strategy as the opponent. Thus, ceteris paribus, strategies that impose negative (positive) externalities are more (less) likely to be selected by evolution, possibly leading to inefficient outcomes.

**Figure 4:**
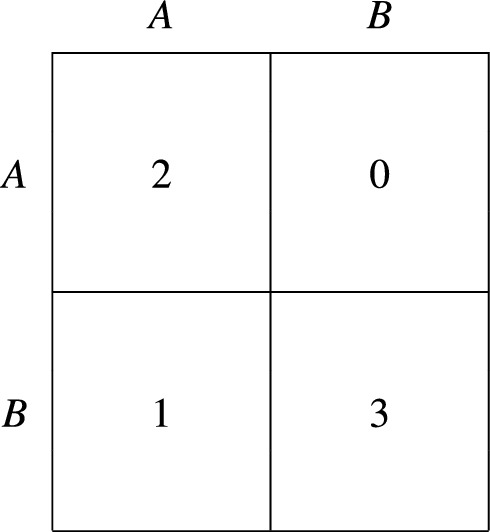
A game where PIID selects an efficient outcome.

However, it is worth noting that PIID do not necessarily lead to inferior outcomes as compared to other evolutionary dynamics. The simple example of Fig. 4 shows this. For instance, under Pairwise Proportional Imitation^12,22^, if the fraction of agents playing *A* is sufficiently large, then the system will move to the state where the whole population plays *A*—which, however, is Pareto dominated by everyone playing *B*. Conversely, under PIID, the system always moves to the state where everyone plays *B* (since *B* is supreme). This result holds in general, i.e., even without the additional assumptions required to represent evolution by means of the modified replicator equation.

Overall, our findings provide a case for why individual behaviors may direct evolution towards outcomes that do not meet Pareto efficiency and strategy dominance criteria. Rather, our dynamics depend on which strategy, if any, is supreme, i.e. systematically outperforms other strategies when these are chosen by one’s opponents. This implies that the outcome of evolution can be either very undesirable or very desirable, depending on how large the payoff from the supreme strategy is when this strategy is chosen by everyone in the population. Moreover, since the structure of interactions plays no role in determining which strategy is supreme, the long-run equilibrium selected by PIID is not affected by institutions and other factors that influence how agents interact, such as those generating assortment.

These results may help explain previous findings in the literature showing that local interactions favor the evolution of cooperation when considering death-birth processes, but not when considering birth-death processes^47^. This can be interpreted as originating from differences in the relation between the interaction structure and agents’ reference groups: death-birth processes assume a distinction between matching and comparisons, whereas birth-death processes make them coincide (as is the case in our model), thereby causing cooperation to be selected against in the long run.

We have shown that when applied to the evolution of behavior, pairwise interact-and-imitate dynamics lead to clear-cut and sometimes surprising results in a variety of games. However, not all classes of games are suited to our revision protocol; in this paper we have considered only symmetric games, leaving aside those cases where agents can choose among different strategies or have different payoff functions. When agents’ strategy sets differ from one another, it does not seem very reasonable to assume that choices are updated according to a pairwise imitative rule based on payoff differentials. Nevertheless, we believe that an imitative protocol like ours may still be applied in a meaningful way to those cases in which agents have the same strategy set but differ in some other respect. For instance, in a setting where agents differ in wealth, a poor individual may be driven to imitate the strategy chosen by a rich individual earning a high payoff, even if this is due to differences in wealth rather than in strategy.

An extension of the model developed here would be to consider the case of a finite population of agents. This would facilitate comparisons with some of the literature^32^, but would come at the cost of hindering the analysis when introducing payoff uncertainty and studying how PIID relate to replicator dynamics. Another extension would be to move from two-player to *n*-player symmetric games, which would require defining the class of Groupwise Interact-and-Imitate Dynamics and adjusting the notion of supremacy to consider the relative performance of a strategy towards profiles of others’ strategies.

Finally, a question that may be worthy of further investigation is how the dynamics will behave when no supreme strategy exists. To answer this question, one may define a binary relation $\succcurlyeq$ such that $i \succcurlyeq j$ if and only if $F(i,j) \ge F(j,i)$. One may then define $\succcurlyeq ^*$ as the transitive closure of $\succcurlyeq$, and let ${\mathcal{S}}:= \{i \in S: i \succcurlyeq ^* j\ \forall \ j \in S \}$ be set of supremal strategies. Our conjecture is that, under PIID, all strategies that do not belong to ${\mathcal{S}}$ will die out independently of the structure of interactions; however, the precise characterization of limit sets may depend on details of the payoff structure, the interaction structure, and the revision protocol.

## Methods

### Lyapunov’s method

To prove Proposition 1 we use Lyapunov’s second method for global stability. We want to show that $f: X_{x^*} \rightarrow [0,1)$, with

$$\begin{aligned} f(x) = 1 - x_i, \end{aligned}$$

is a strict Lyapunov function. It is easy to see that *f* is of class $C^1$, that $f(x) > 0$ for every $x \in X_{x^*} \setminus \{x^*\}$, and that $f(x^*) = 0$. We are left to show that (i) $\dot{f}(x^*) = 0$ and (ii) $\dot{f}(x) < 0$ for every $x \in X_{x^*} \setminus \{x^*\}$. Taking the time derivative of *f* and using Definition 1, we can write:

$$\begin{aligned} \dot{f}(x) = \frac{\partial f(x)}{\partial x_i} \dot{x}_i = - \sum _{j \in S} p_{ij}(x) \phi _{ij}(x). \end{aligned}$$

We observe that $p_{ij}(x^*) = 0$ for every $j \in S$, which implies $\dot{f}(x^*) = 0$. For every $x \in X_{x^*} \setminus \{x^*\}$, there exists $k \ne i$ such that $x_k >0$. Since $x_i >0$ and $x_k >0$, we therefore have that $p_{ik}(x) >0$. Moreover, since $F(i,j)>F(j,i)$ for every $j \in S \setminus \{i\}$, we have that $\phi _{ik}(x)>0$ by Assumption 1. To see that $\dot{f}(x) < 0$ for every $x \in X_{x^*} \setminus \{x^*\}$, we finally note that $p_{ij}(x)$ is always non-negative and that $\phi _{ij}(x)$ is positive for all $j \in S \setminus \{i\}$ by the definition of supremacy and Assumption 1.

### Supremacy under uncertainty

So far we have only considered games in which payoffs are not subject to any uncertainty in their realization. Here we extend our analysis by allowing for this possibility, which is relevant for a variety of applications (e.g., the Hawk–Dove and Hawk–Dove–Bourgeois games).

Let $\Omega$ be a finite set of states of the world and $q_{\omega }$ be the probability of state $\omega \in \Omega$ occurring. We write $F(i,j | \omega )$ to denote the payoff received by an agent playing strategy *i* against an opponent playing *j* when state of the world $\omega$ occurs. For every $x \in X$ and every $\omega \in \Omega$, let $\phi (x|\omega )$ be the $n \times n$-matrix with typical element $\phi _{ij}(x|\omega )$, the latter being the net inflow from *j* to *i* when the population state is *x*, the state of the world is $\omega$, and an *ij* pair is formed.

We replace Assumption 1 with the following.

#### **Assumption 2**

For every $x \in X$ and every $\omega \in \Omega$, $\phi _{ij}(x|\omega ) > 0$ if $F(i,j|\omega ) > F(j,i|\omega )$.

We also assume that, at any point in time, the net population inflow from *j* to *i* is obtained by averaging $\phi _{ij}$ over the set of states of the world, i.e.:

3 $$\begin{aligned} \phi _{ij}(x) = \sum _{\omega \in \Omega } q_{\omega } \phi _{ij}(x|\omega ). \end{aligned}$$

The convergence result of Proposition 1 can be extended to the case of uncertainty if we define supremacy as follows.

#### **Definition 3**

(*Supremacy under uncertainty*) In the presence of uncertainty, strategy $i\in S$ is supreme if $F(i,j|\omega ) > F(j,i|\omega )$ for every $\omega \in \Omega$ and every $j \in S \setminus \{i\}$.

To see that the result holds, note that if strategy *i* is supreme by Definition 3 and Assumption 2 holds, then $\phi _{ij}(x) > 0$ for every $j \in S \setminus \{i\}$ and every $x \in X$. Moreover, $\phi$ is continuous in *X* if $\phi (\cdot ,\omega )$ is continuous in *X* for every $\omega \in \Omega$. By a reasoning analogous to that used in the proof of Proposition 1, we therefore have that state $\left\{ x \in X : x_i = 1 \right\}$ is globally asymptotically stable for the dynamical system with state space $X_{x^*}$ and PIID as equation of motion.

A less restrictive definition of supremacy under uncertainty is given below.

#### **Definition 4**

(*Weak supremacy under uncertainty*) In the presence of uncertainty, strategy $i\in S$ is weakly supreme if $F(i,j|\omega ) \ge F(j,i|\omega )$ for every $\omega \in \Omega$ and every $j \in S \setminus \{i\}$, and if for every $j \in S \setminus \{i\}$ there exists $\hat{\omega } \in \Omega$ such that $F(i,j|\hat{\omega })>F(j,i|\hat{\omega })$.

Under the conditions of Definition 4, our convergence result holds if we strengthen Assumption 2 as follows.

#### **Assumption 3**

For every $x \in X$ and every $\omega \in \Omega$, $\phi _{ij}(x|\omega ) > 0$ if and only if $F(i,j|\omega ) > F(j,i|\omega )$.

Here the ‘only if’ is required to deal with those states of the world where $F(i,j|\omega ) = F(j,i|\omega )$, which are not ruled out by Definition 4.

An even weaker definition of supremacy can be given when focusing on a specific $\phi$. For instance, consider the case where the probability that agents have to imitate the opponent is proportional to the difference in their payoffs if the opponent’s payoff exceeds their own, and is zero otherwise. Under this protocol, letting the expected payoff from playing *i* against *j* be $\mathbb {E} \left[ F(i,j) \right] = \sum _{\omega \in \Omega } q_{\omega } F(i,j|\omega )$, we can define the following.

#### **Definition 5**

(*Supremacy in expectation under uncertainty*) In the presence of uncertainty, strategy $i\in S$ is supreme in expectation if $\mathbb {E} \left[ F(i,j) \right] > \mathbb {E} \left[ F(j,i) \right]$ for every $j \in S \setminus \{i\}$.

It can now be seen that:

$$\begin{aligned} \phi _{ij}(x)> 0 \Leftrightarrow \sum _{\omega \in \Omega } q_w \left[ F(i,j \mid \omega ) - F(j,i \mid \omega )\right]> 0 \Leftrightarrow \mathbb {E} \left[ F(i,j) \right] > \mathbb {E} \left[ F(j,i) \right] , \end{aligned}$$

meaning that the net population inflow from *j* to *i* is positive if and only if *i* is supreme in expectation. This suffices to replicate the result of Proposition 1.

### Heterogeneous revision protocols

Although we believe the interact-and-imitate protocol to be reasonable in many circumstances, it may well be the case that agents also rely on other revision protocols occasionally. If our results crucially hinged on the assumption that agents always follow the interact-and-imitate rule, they would be of little interest.

Let the set of possible states of the world be $\Omega = \{\omega _1, \omega _2\}$. Suppose that agents follow the pairwise interact-and-imitate protocol in state $\omega _1$ and a different revision protocol in state $\omega _2$. Now let us define a continuous function $\rho : X \times \mathscr {P} \rightarrow [-1,1]$, where $\rho _{ij}(x) \in [-1,1]$ is the probability that an *ij* pair will turn into an *ii* pair minus the probability that it will turn into a *jj* pair, conditional on the population state being *x*, an *ij* pair being formed, and the state of the world being $\omega _2$. Note that $\rho$ may also reflect the fact that members of a pair interact after, rather than before, having updated their strategies, in which case the interact-and-imitate protocol cannot be applied.

We define:

4 $$\begin{aligned} {\hat{\phi}}_{ij}(x) :=(1-\varepsilon )\phi _{ij}(x) + \varepsilon \rho _{ij}(x), \end{aligned}$$

with $\varepsilon \in \left( 0, 1 \right)$. The equation of motion for this dynamical system is the following.

#### **Definition 6**

(*Quasi pairwise interact-and-imitate dynamics—QPIID*) For every $x \in X$ and every $i \in S$:

5 $$\begin{aligned} \dot{x}_i = \sum _{j \in S} p_{ij}(x) {\hat{\phi}}_{ij}(x). \end{aligned}$$

Note that Assumption 1 concerns $\phi$ only, and that we have no analogous assumption for $\rho$. This notwithstanding, a convergence result in the spirit of Proposition 1 can be obtained if agents follow the $\rho$ protocol rarely enough.

#### **Proposition 3**

*If* $i \varepsilon S$ *is supreme, then there exists* $\bar{\varepsilon }>0$ *such that, for every* $\varepsilon \in (0,\bar{\varepsilon })$, *state* $x^* \equiv \left\{ x \in X : x_i = 1 \right\}$ *is globally asymptotically stable for the dynamical system with state space* $X_{x^*}$ *and QPIID as equation of motion*.

We want to show that if $F(i,j)>F(j,i)$ for every $j \in S \setminus \{i\}$, then there exists $\bar{\varepsilon }>0$ such that, for every $\varepsilon \in (0,\bar{\varepsilon })$, ${\hat{\phi}}(x)>0$ for every $x \in X$. Moreover, since both $\phi$ and $\rho$ are continuous in *X*, ${\hat{\phi}}$ is continuous in *X* as well. As a consequence, the statement in Proposition 3 can be proven by replicating the argument used in the proof of Proposition 1.

We consider the worst case to have ${\hat{\phi}}(x)$ positive. For every $j \in S \setminus \{i\}$, we have that $\bar{\phi }_{ij} := \min _{x \in X} \phi _{ij}(x)$ exists, since $\phi _{ij}$ is a continuous function on a compact set. Moreover, by continuity of $\phi _{ij}$ and noting that $\phi _{ij}(x)>0$ due to $F(i,j)>F(j,i)$ and Assumption 1, we also have that $\bar{\phi }_{ij} > 0$. We define $\bar{\phi }_i := \min _{j \ne i} \bar{\phi }_{ij}$, and note that $\bar{\phi }_i>0$. Also, we define:

6 $$\begin{aligned} \bar{\varepsilon } :=\frac{\bar{\phi }_i}{1+\bar{\phi }_i}. \end{aligned}$$

Since $\rho _{ij}$ cannot be smaller than $-1$, if $\varepsilon < \bar{\varepsilon }$ then we have that ${\hat{\phi}}(x)>0$ for every $x \in X$, as can be easily checked from (4).

We stress that the bound on $\varepsilon$ given in (6) is independent of which $\rho$ protocol is being considered. A more precise value for the bound can be obtained by making specific assumptions about the strategy revision process. For example, suppose that in state of the world $\omega _1$, agents imitate the opponent with unit probability whenever the latter receives a higher payoff than they do, so that $\phi _{ij} = 1$ if $F(i,j) > F(j,i)$. In this case, we can have the maximum possible amount of heterogeneity in revision protocols (i.e., $\bar{\varepsilon } = 1/2$) and still have global asymptotic stability of the state in which the whole population chooses the supreme strategy.
